# Methotrexate and Cytarabine—Loaded Nanocarriers for Multidrug Cancer Therapy. Spectroscopic Study

**DOI:** 10.3390/molecules21121689

**Published:** 2016-12-08

**Authors:** Danuta Pentak, Violetta Kozik, Andrzej Bąk, Paulina Dybał, Aleksander Sochanik, Josef Jampilek

**Affiliations:** 1 Department of Materials Chemistry and Chemical Technology, Institute of Chemistry, University of Silesia, 40-006 Katowice, Poland; 2 Department of Synthesis Chemistry, Institute of Chemistry, University of Silesia, 40-006 Katowice, Poland; 3 Department of Organic Chemistry, Institute of Chemistry, University of Silesia, 40-006 Katowice, Poland; andrzej.bak@us.edu.pl (A.B.); pdybal@us.edu.pl (P.D.); 4 Center for Translational Research and Molecular Biology of Cancer, Maria Skłodowska-Curie Memorial Cancer Center and Institute of Oncology, Gliwice Branch, 44-100 Gliwice, Poland; asochanik@gmail.com; 5 Department of Pharmaceutical Chemistry, Faculty of Pharmacy, Comenius University, 832 32 Bratislava, Slovakia

**Keywords:** delivery systems, cytarabine, methotrexate

## Abstract

Determining the properties of nanoparticles obtained by novel methods and defining the scope of their application as drug carriers has important practical significance. This article presents the pioneering studies concerning high degree incorporation of cytarabine (AraC) and methotrexate (MTX) into liposome vesicles. The main focus of this study were cytarabine-methotrexate-dipalmitoylphosphatidylcholine (DPPC) interactions observed in the gel and fluid phases of DPPC bilayers. The proposed new method of use the Transmittance_2919/2850_ ratio presented in our research is sensitive to subtle changes in conformational order resulting from rotations, kinks and bends of the lipid chains. The transition temperatures characterized by Fourier Transform Infrared Spectroscopy (FT-IR) were consistent with the results obtained by Differential Scanning Calorimetry (DSC). Transmission Electron Microscopy (TEM) was used in order to determine the size and shape of the liposomes obtained. The mutual interactions occurring between the drugs studied and the phospholipids were analyzed using the Nuclear Magnetic Resonance (NMR).

## 1. Introduction

Important issues concerning transport of therapeutic substances, drug carriers and therapeutic delivery systems (TDS) include form of the drug, its formulation, structure and/or technology applied. The paramount objective is to modify and improve bio-availability of the therapeutic effect of the medicinal agent, and/or to minimize side effects by influencing its pharmacokinetic-pharmacological parameters. The premises of modern pharmaceutical technology are associated with a desire to achieve optimum therapeutic drug concentration at its site of action, usually by modifying the process of drug release. It has been estimated that approximately 40%–50% of new therapeutic substances would require the use of TDS [1].

The developments in material chemistry research, especially nanotechnology, have led to the acquisition of new biocompatible materials that can be used in formulating novel drug carriers. The results of these discoveries have translated into a number of medicinal TDS which have been registered by the FDA (US Food and Drug Administration) over the past few decades. Most TDS are polymer-drug conjugates, where the polymer unit is made up of polyethylene glycol or copolymers of lactic acid and glycolic acid. Nonetheless, many drugs can also be transported by lipid-based structures, i.e., micelles and liposomes. Liposomes are spherical structures formed by a single lipid bilayer or multiple, concentric bilayers which enclose part of the aqueous medium in which they are suspended. This bilaminar structure is made up of amphiphilic phospholipids arranged in two adjacent layers. The hydrophilic portions of the lipids, forming the double shell of the liposome, are positioned towards the water phase, while the nonpolar lipophilic fatty acid chains of both lipid layers are directed towards each other to form an inner hydrophobic layer.

A notable feature of liposomes is their biocompatibility. Liposomal membrane composition similar to natural components of the cell membrane causes the liposomes to be non-toxic and biodegradable. Another important feature is their size. Depending on the method of liposome preparation, they may vary in size from dozens of nanometers to several micrometers, with a membrane thickness of approximately 4 nm. The size of liposomes is crucial for rate of their removal from the bloodstream by macrophages of the MPS (Mononuclear Phagocyte System). Shortly after injection into the blood circulatory system, liposomes start interacting with plasma proteins (opsonins), which, when adsorbed on the surface of liposomes, lead to their removal by the MPS. Liposomes with a diameter >100 nm are caught by the MPS much quicker and easier. On the other hand, small vesicles (<100 nm) have a longer circulation time in the bloodstream [2]. The use of small liposomes with a diameter of about 100 nm allows them to penetrate through the walls of blood vessels. This is particularly important at sites of inflammation, wherein the vessel walls exhibit considerable spaces between cells. The gaps are from 100 to 200 times larger than those occurring in healthy blood vessels [3,4]. Through these gaps, liposomes can easily penetrate and concentrate themselves at the site of inflammation. In the case of healthy tissues, vascular walls are poorly permeable, and the spaces between the cells range between approximately 20 to 40 nm. As a result, liposomes “do not exit” the bloodstream and remain there until they reach their destination or are removed. Liposomes remaining in the bloodstream for an extended period of time are biodegraded gradually, thereby providing a stable level of drug activity. This “passive targeting” is extensively used by nanotechnology-based delivery systems, including liposomes for drug delivery and treatment of tumors, bacterial or viral infections and many other diseases.

**Figure 1:**
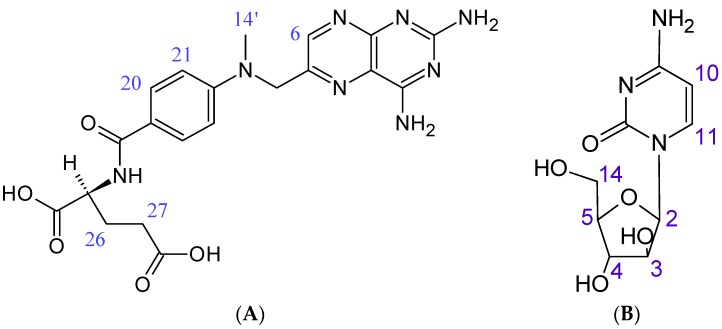
Chemical structures of (**A**) methotrexate; (**B**) cytarabine.

This article presents the pioneering studies concerning high degree incorporation of cytarabine (AraC) and methotrexate (MTX) (Figure 1) simultaneously into liposome vesicles obtained by a modified reverse-phase evaporation method (mREV).

Based on the clinical studies, there is a lot of information that contain therapeutic protocols concerning the use of cytarabine and methotrexate together (cyclophosphamide, vincristine, methotrexate, leucovorin, cytarabine (COMLA), doxorubicin, methotrexate, vincristine, prednisone, leucovorin, cytarabine, cyclophosphamide, etoposide (AMOPLACE), carmustine, vincristine, cytarabine, methotrexate (BVAM), fluorouracil, methotrexate, cytarabine, cyclophosphamide, doxorubicin, vincristine, prednisone (F-MACHOP), cyclophosphamide, high-dose methotrexate, high-dose cytarabine (HIC-COM), methotrexate, vincristine, prednisone, cytarabine, cyclophosphamide, etoposide (MOPLACE)). Until recently, commonly used monotherapy was increasingly being replaced by a combination and targeted therapy. Targeted therapy by the use of compounds that inhibit specific target molecules can provide new perspectives on the treatment of cancer. The use of multidrug therapy, in contrast to conventional chemotherapy, has many advantages. Cancer cells are attacked by multiple drugs that disrupt different stages of the cell reproduction cycle. For instance, minimization of side effects is another goal of combination therapy. The use of liposomes in the transport of drugs can minimize the occurrence of multidrug therapy side effects. It is noteworthy that the presented study was mainly focused on cytarabine-methotrexate-dipalmitoylphosphatidylcholine interactions in the 20–47 °C temperature range, i.e., in the gel (P_β’_) and fluid (L_α_) phases of dipalmitoylphosphatidylcholine (DPPC) bilayers.

The knowledge of drug carriers’ thermotropic properties provides a lot of practical information at the primary studies stage. The extent and rate of drug release arising from thermotropic properties determine the practical application of newly obtained structures. The proposed new method of using the Transmittance_2919/2850_ ratio presented in our research is sensitive to subtle changes in conformational order resulting from rotations, kinks and bends of the lipid chains. The transition temperatures characterized by Fourier Transform Infrared Spectroscopy (FT-IR) were consistent with the results obtained by Differential Scanning Calorimetry (DSC). Transmission Electron Microscopy (TEM) was employed in order to determine the size and shape of the liposomes obtained. Mutual interactions occurring between both studied drugs and the phospholipids were analyzed using NMR.

The results presented herein are a continuation of research on the encapsulation of two drugs into liposomes, one of which has been cytarabine [5,6,7,8]. Cytarabine is a chemotherapeutic used to treat leukemia and lymphoma. The other drug studied herein, methotrexate, has been employed to treat breast, head and neck, lung and bladder neoplasms. However, the associated toxicity and side effects of both warrant evaluation of novel efficient delivery systems to improve the therapeutic efficiency of such drugs [9].

## 2. Results and Discussion

### 2.1. Liposome Properties

**Figure 2:**
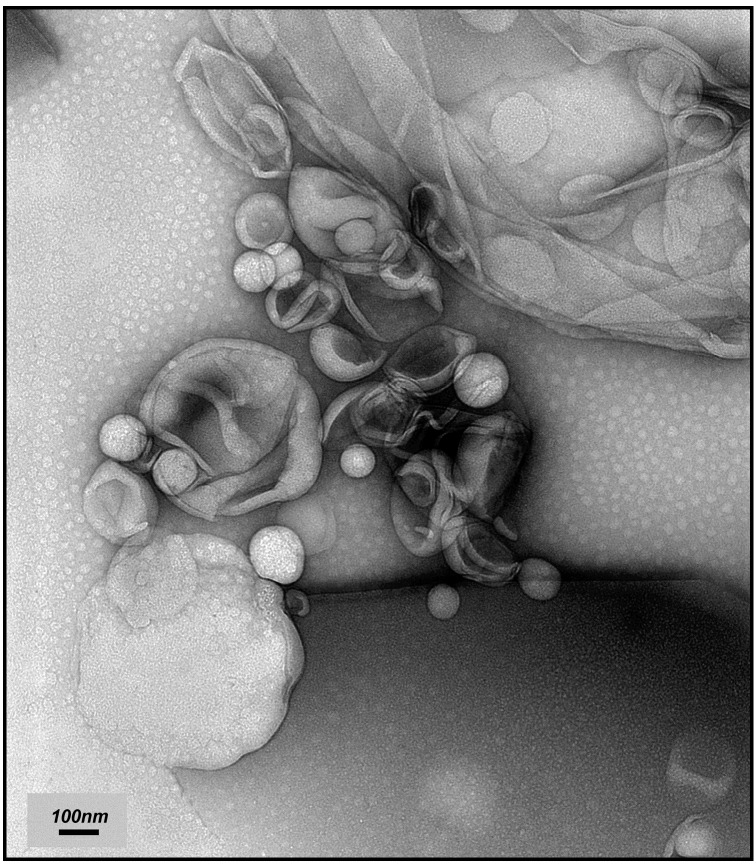
Transmission electron micrographs of liposomes containing cytarabine and methotrexate (L_DPPC/AraC/MTX_) obtained by the modified reverse-phase evaporation method (mREV), negatively stained (2% uranyl acetate solution) on a carbon-coated copper grid, magnification: 50,000 times; at room temperature.

Several imaging techniques (SEM—Scanning Electron Microscopy, TEM, AFM—Atomic Force Microscopy, Confocal Microscopy) can be used to determine the size and shape of liposomes. Ruozi et al. suggested freeze–fracture to be the optimal method for studying biological samples in this context [10]. In the present study, we used a simpler and faster imaging procedure based on TEM. In this method, liposomes are embedded in a suitable electron-dense material providing high contrast and good reproducibility. Uranyl acetate (2%), which was used herein, penetrates only slightly into the interior of liposomes, and mostly binds to the phosphate groups of the external lipid layer. Thus, it allows for an assessment of the shape and size of the resulting liposomes (Figure 2). Based on these results, it was found that the L_DPPC/AraC/MTX_ liposomes obtained via the mREV method are characterized by a regular spherical shape and a size of ≤120 nm.

**Figure 3:**
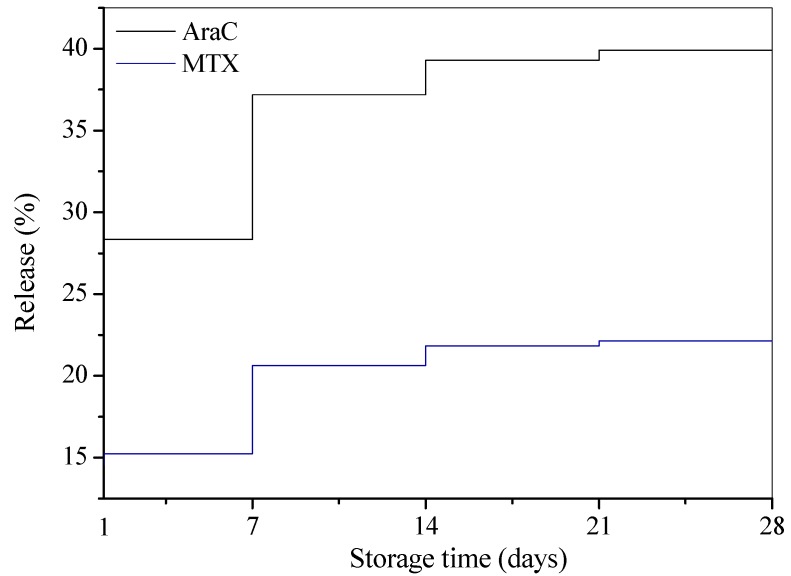
In vitro cytarabine (AraC) and methotrexate (MTX) release from the L_DPPC/AraC/MTX_ liposomes stored at +4 °C for a period of four weeks.

The drugs used in the preparation of the liposomes were used at 1:1 molar ratio. The determination of the encapsulated cytarabine and methotrexate was carried out according to the method described by Kaiser et al. [11]. The encapsulation efficiency was determined as the mass ratio between the amount of the drug incorporated in liposomes and the ratio used in the liposome preparation. The encapsulation efficiencies of AraC and MTX in L_DPPC/AraC/MTX_ were found to be 86.3% (AraC) and 86% (MTX). In order to determine the stability of the liposomal preparations, the percentage of cytarabine and methotrexate release from the liposomes was monitored for several weeks. The drug release measurements were performed according to the method reported by Jin et al. [12]. The results of the stability study, based on weekly determinations of the release rate, are shown in Figure 3.

The results obtained for a four-week-long incubation of liposomes at +4 °C have demonstrated that the simultaneous incorporation of cytarabine and methotrexate into liposomes (molar ratio 1:1) takes place on a competitive basis, and as the result of this process, double release of cytarabine from liposomes occurs compared to methotrexate.

### 2.2. Thermodynamic Properties of Phase Transition

**Figure 4:**
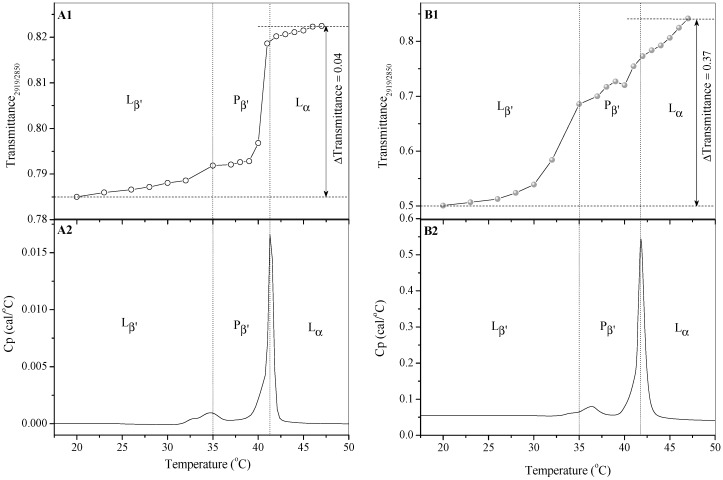
Transmittance_2919/2850_ ratio (**A1**,**B1**) vs. temperature and Differential Scanning Calorimetry (**A2**,**B2**) plots of (**A**) control DPPC liposomes and (**B**) DPPC liposomes containing cytarabine and methotrexate. L_β__’_—tilted gel phase; P_β__’_—ripple gel phase; L_α_—liquid crystalline phase; Cp—heat capacity at constant pressure.

Knowledge of the size and shape of liposomal structures, made possible by the applied TEM method, has been extremely important at the current stage of research on the proposed liposomal drug formulation. Other important aspects of this basic research involve recognition of thermodynamic changes in phospholipid membranes and interactions between the incorporated drugs and phospholipid molecules. The Differential Scanning Calorimetry (DSC) and FT-IR methods enabled a determination of chain fluidity and mobility alterations, and ultimately permitted correlation of the results with the spectroscopic structural modifications on a molecular level. Figure 4 represents changes in the Transmittance_2919/2850_ ratio of L_DPPC_ and L_DPPC/AraC/MTX_ liposomes and calorimetric scans from L_DPPC_ and L_DPPC/AraC/MTX_ liposomes.

The dynamics and structural changes occurring in the phospholipid membrane resulted in observing two transition phases. The first one, known as the pre-transition phase (Tp ~ 35 °C) is the transition from L_β’_ phase to P_β’_ phase. The second, called the main transition phase (Tc ~ 41 °C), is the transition from P_β’_ phase to L_α_ phase. Below the pre-transition temperature (Tp), the lipid chains are in all-*trans* conformation [13]. Tristram-Nagle et al. concluded that, in L_β’_ phase, the lipid chains are tilted with respect to the normal membrane at an angle of about 32° [14]. Above the temperature of the pre-transition phase, the acyl chains remain in the folded gel phase.

FT-IR spectra of blank (L_DPPC_) liposomes and of liposomes containing cytarabine and methotrexate (L_DPPC/AraC/MTX_) were obtained at 20–47 °C and were recorded in the 3000–800 cm^−1^ range. When analyzing the FT-IR spectra of the lipid samples, it is easy to see that the methylene stretching bands in the 3000 cm^−1^–2850 cm^−1^ region are the most intense ones in the whole spectrum. The symmetric stretching modes in the methylene groups (CH_2_) allow for an investigation of the main phase transition temperature (Tc). The values for the main phase transition temperature Tc obtained by FT-IR and DSC methods are comparable (Table 1). For the referenced liposomes, the temperature Tc obtained by DSC is 41.30 °C ± 0.08, and that obtained by FT-IR is 41.28 °C ± 0.06. After incorporation of the drugs, this temperature increases slightly as a result of changes in the packing of molecules in the film as well as changes in the conformation of lipids. The incorporation of drugs also has a significant impact on the temperature of the pre-transition phase (Tp). The results of the Maghraby et al. study concerning the interaction of drugs having differing degrees of solubility in water with phospholipid membranes suggest that the pre-transition phase is the result of, first, the rotation of the polar part of the phospholipid, and, second, conformational changes of the phospholipid membrane [15]. Thus, any alteration of the membrane composition with additional ingredients will ultimately affect the temperature of the pre-transition phase. The incorporation of cytarabine and methotrexate into phospholipid membranes resulted in the increase of Tp temperature by nearly 2 °C. This indicates that the analyzed drugs penetrate only partially into the bilayer (into the lipophilic region) and always remain in direct contact with the polar portion of dipalmitoylphosphatidylcholine.

The FT-IR method also allows estimating the degree of membrane ordering. The use of the intensity ratio of two bands as an order parameter (I_2850_/I_2880_) was first proposed by Levin and Lewis in 1990 [16]. Since then as reported by Potamitis and co-workers [17], these bands have been commonly used to monitor changes in the lateral packing properties and mobility of the lipid chain in both gel and liquid crystalline bilayer systems. The Transmittance_2919/2850_ ratio presented in our research, similarly to the intensity ratio I_2850_/I_2880_ proposed by Levin and Lewis, is sensitive to subtle changes in conformational order resulting from rotations, kinks and bends of the lipid chains. The isobaric curve derived from changes in the position of symmetrical and asymmetrical stretching vibration bands shows three temperature ranges for reference liposomes as well as for liposomes with incorporated cytarabine and methotrexate (Figure 4A1,B1). The phase transitions determined by this method are consistent with the data received using the DSC method. It is also clear that cytarabine and methotrexate increase the degree of the membrane ordering. The degree of membrane ordering is 10-fold less for the reference liposomes as compared to the L_DPPC/AraC/MTX_ (ΔTransmittance_2919/2850_ increases from 0.04 to 0.37).

Observation on the basis of ΔTransmittance_2919/2850_ growth in the degree of membrane order after incorporation of cytarabine and methotrexate is comparable to the increase in the value of the parameter S, which has been determined by EPR spectroscopy used for cholesterol incorporated into liposomes [18,19]. As can be seen from Figure 4A1,B1, cytarabine and methotrexate induce the greatest changes in the conformation of lipids already in the tilted gel phase.

### 2.3. DPPC Liposomes Containing Cytarabine and Methotrexate: Studies by FT-IR and NMR Spectroscopy

**Figure 5:**
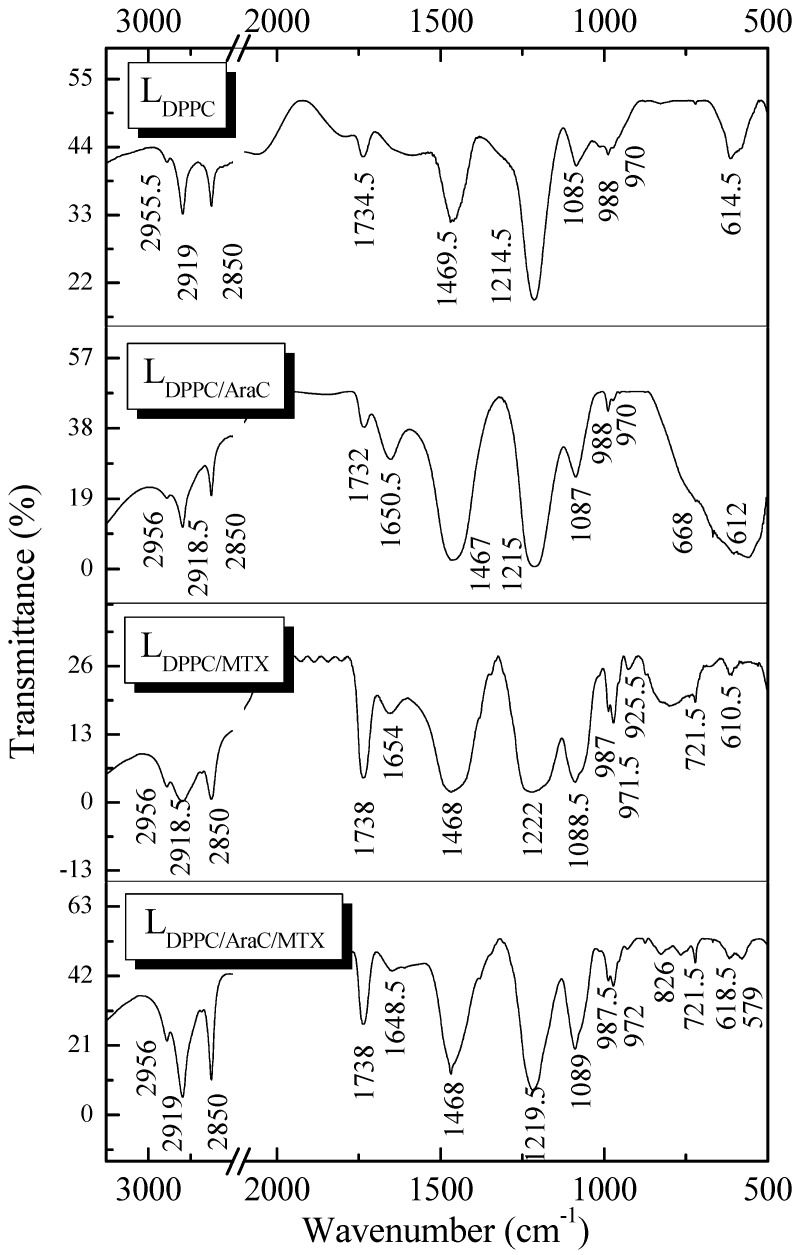
FT-IR spectra of control L_DPPC_ liposomes and liposomes containing cytarabine and methotrexate.

As presented in Figure 5, changes in the FT-IR spectrum are visible in the entire spectral region. The changes observed for each band, resulting from the incorporation of the analyzed drugs, are shown in Table 2. The principal band between 3000 cm^−1^ and 2800 cm^−1^ represents the C–H stretching modes with the maxima at 2919 cm^−1^ and at 2850 cm^−1^ corresponding to antisymmetric and symmetric stretching in the CH_2_ groups of alkyl chains, respectively. No contribution from the antisymmetric stretching vibration in the CH_3_ groups at 2956 cm^−1^ was observed. In the spectral region between 1800 cm^−1^ and 1500 cm^−1^, the stretching vibrations of the C–O groups were observed. This band is attributed to the stretching vibration of the C–O group in the ester band. As presented in Table 2, spectral changes in this region arise both from the incorporation of cytarabine as well as methotrexate. The biggest inductive influence of oxygen in the ester fragment of the DPPC molecule causes the increase of frequency of the C–O group (1734.5→1738 cm^−1^) in liposomes containing methotrexate.

The effects include changes in electron density and bond lengths. A strong wide band in the spectral region of ~1214 cm^−1^ was also observed. This band is attributed to the asymmetrical stretching vibrations of the –PO_2_^−^ group in DPPC. The changes in the area of the phosphate group were weak for L_DPPC/AraC_ liposomes. Much more significant (1214.5→1222 cm^−1^) changes in the analyzed area of the spectrum were caused by the incorporation of methotrexate into phospholipid membranes. The vibrations of polar head groups are represented by symmetric PO_2_^−^ stretching (~1085 cm^−1^) and antisymmetric N^+^–CH_3_ stretching (~970 cm^−1^) vibrations. The simultaneous incorporation of cytarabine and methotrexate into liposomes increases the frequency of vibration of the PO_2_^−^ by 4 cm^−1^. The observed changes in the polar head groups area, resulting from the incorporation of drugs, also confirm the changes in temperature of the pre-transition phase (Table 1). It is also known that the molecular reorientation of polar head groups from the horizontal to the vertical position is strongly endothermic [20].

**Figure 6:**
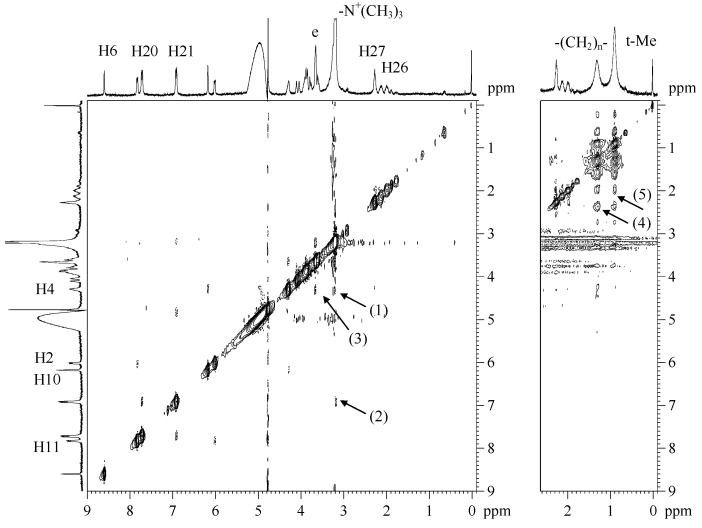
2D NOESY spectra of L_DPPC/AraC/MTX_ liposomes obtained at 25 °C (**left** panel) and at 47 °C (**right** panel).

As expected, NMR spectroscopy has confirmed the presence of interactions between the analyzed drugs and the phospholipids forming the liposomal membrane (Figure 6). Schematically, the liposomal membrane can be divided into three areas: the polar area, the hydrogen belt and the hydrophobic core. Partial positioning of cytarabine and methotrexate in the polar area of the membrane has been confirmed by correlation signals between H4 cytarabine protons and protons of the quaternary ammonium group (1), and between the protons of the quaternary ammonium group and the H21 methotrexate protons (2). Moreover, a cross peak between H4 (Figure 1) cytarabine protons and dipalmitoylphosphatidylcholine protons was observed (3). In order to facilitate the interpretation of the 2D NOESY spectra, the structure and ^1^H-NMR spectrum of dipalmitoylphosphatidylcholine has been added in Supplementary Figure S1.

With reference to our previous research on the positioning of cytarabine in the liposome membrane [7,8], it may be concluded that the sugar residue of cytarabine, which contains hydroxyl groups, orients itself towards the lipid–water interface, and the pyrimidine ring penetrates into the glycerol backbone area. Raising the analytical temperature to 47 °C (Figure 6, right panel) confirmed the location of methotrexate in the hydrophobic area of the membrane. In the 2D NOESY spectrum at 47 °C, NOEs are seen between the H26 and H27 protons of methotrexate (Figure 1) and the (CH_2_)_n_ protons of the alkyl chain of the lipid bilayer (4) and the terminal methylene group (5). Given the results obtained by FT-IR, it can be concluded that the methotrexate molecules are located perpendicularly to the surface of the membrane, passing through the hydrophilic area up to the hydrophobic portion of the membrane.

The overall conclusion is that the simultaneous use of FT-IR and NMR spectroscopy allowed us to determine the location of drugs in the liposomal membrane and the interaction between cytarabine, methotrexate and DPPC. The complementarity of used techniques confirmed the changes observed in the spectra such as NMR and FT-IR.

## 3. Materials and Methods

### 3.1. Materials

l-α-phosphatidylcholine dipalmitoyl (1,2-dihexadecanoyl-*sn*-glycerol-3-phosphocholine) (DPPC, purity 99%), 1-β-d-arabinofuranosylcytosine (cytarabine, AraC), dl-4-Amino-*N*^10^-methylpteroylglutamic acid (methotrexate, MTX) were purchased from Sigma-Aldrich, Schnelldorf, Germany. Chloroform, dichloromethane, sodium hydroxide and phosphate buffered saline (PBS buffer, pH 7.4: K_2_HPO_4_, NaH_2_PO_4_) were supplied by POCH, Gliwice, Poland. Deuterium oxide (D_2_O) 99%, chloroform-*d* 99%, stabilized with Ag and sodium 4,4-dimethyl-4-silapentane sulfonate (DSS) were purchased from ARMAR Chemicals, Döttingen, Switzerland.

### 3.2. Methods

#### 3.2.1. Liposome Preparation

Small (diameter ≤120 nm) liposomes (L_DPPC_; L_DPPC/AraC/MTX_) were obtained by the modified reverse-phase evaporation method (mREV) [5] using a DPPC:drug:drug molar ratio of 30:1:1. The lipid dispersion (final lipid concentration 2.64 × 10^−2^ M) was mixed with 0.175 mL of 5 × 10^−3^ M AraC and 0.175 mL of 5 × 10^−3^ M MTX. A 2 mL PBS buffer (pH = 7.4) and a 4 mL organic solution prepared from methylene chloride and chloroform were applied. The preparation process was carried out at 44 °C. The average time of liposome preparation did not exceed 12 min. The liposome entrapped analysed drugs were separated from the free drugs by dialysis in Float-A-Lyzer G2 (Spectra/Por) (Spectrum Laboratories, Inc., Rancho Dominguez, CA, USA) tubing with several changes of buffer at 4 °C.

#### 3.2.2. Solution and Sample Preparation

A 0.05 M phosphate buffered saline (PBS) solution with a pH adjusted to 7.4 as necessary, to mimic physiological conditions, was prepared by dissolving 3.4836 g of K_2_HPO_4_ and 0.7800 g of NaH_2_PO_4_ in purified water (500 mL total volume). For NMR and FT-IR measurements, deuterated solvents were used. All solutions were prepared in triplicates.

#### 3.2.3. Transmission Electron Microscopy (TEM)

TEM micrographs of liposome preparations before filtration were taken on Philips EM400 (100 keV accelerating voltage, Eindhoven, The Netherlands). Aliquots of liposome phospholipid bilayer solution were placed on copper and carbon-coated grids (300 mesh, Sigma-Aldrich Chemie GmbH, Schnelldorf, Germany). Negative staining with 2% uranyl acetate was applied to enhance image quality. The micrographs of completely dried liposomal bilayers were acquired. A wide range of electron beam of weak intensity (low electron density) was used to prevent sample overheating.

#### 3.2.4. Differential Scanning Calorimetry (DSC)

Differential scanning calorimetry (DSC) scans were performed using the VP DSC ultrasensitive microcalorimeter (MicroCal Inc., Northampton, MA, USA) with 0.5 mL cell volume. Degassing during the calorimetric experiments was prevented by maintaining an additional constant pressure of 1.8 atm over the liquids in the cells. At first, the buffer was placed in both the sample and reference compartments. A DSC curve corresponding to buffer vs. buffer run was used as the instrumental baseline. The calorimetric data were corrected for the calorimetric baseline (by subtracting buffer–buffer scan). Heat capacity vs. temperature profiles were obtained for a scanning rate of 1.0 °C/min over 10–65 °C temperature range. A minimum of at least three heating scans were performed for each analysis and all thermograms were reproducible. The Origin 8.5 software package (OriginLab Corporation, Northampton, MA, USA) was used to evaluate the transition temperatures.

#### 3.2.5. Fourier Transform Infrared Spectroscopy

Fourier transform infrared spectra were obtained using a PerkinElmer Spectrum One FT-IR spectrometer (Waltham, MA, USA) equipped with an IF KT-3 (Photon Institute, Krakow, Poland) automatic temperature controller. Spectra were recorded for both dried and fully hydrated liposome samples. For the dried samples, spectra were recorded at room temperature. The lipid dispersions were placed in a demountable cell between two ZnSe windows separated by a 50 μm thick Teflon spacer. For temperature regulation, the cell was placed in a thermostated jacket with internal temperature measurement. An external water bath was used for temperature control. The temperature was maintained at ±0.1 °C. All spectra were acquired after equilibrating liposome samples for 15 min at each of the desired temperature points (20–47 °C range) using an automatic temperature controller. Data acquisition was performed at intervals of 2 °C. For each spectrum, covering the 3000–800 cm^−1^ region, 10 interferograms were coadded, apodized and Fourier transformed to give a resolution of 2 cm^−1^. FT-IR spectrum for the buffer was obtained under identical instrumental conditions. Peak positions were determined with one significant digit using Spectrum v3.01 spectral processing software (PerkinElmer Inc., Waltham, MA, USA).

#### 3.2.6. NMR Measurements of Liposomes

^1^H-NMR spectra were obtained using 9.4 Tesla Bruker Avance UltraShield (400.130 MHz for ^1^H) (Karlsruhe, Germany) and 5 mm inverse broadband probe (BBI). The water suppression for ^1^H measurements using 3-9-19 pulse sequence with gradients was used. ^1^H-NMR spectra were recorded at a temperature range of 25–47 °C. Sample temperature was controlled by air and monitored by the Bruker thermal control system. The samples were heated at a rate of up to 1.0 °C/min and were left for approximately 15 min to achieve equilibrium, which was monitored based on the free induction decay (FID) signal. The temperature was maintained at ±0.1 °C. Water suppression was obtained by presaturation. For ^1^H-NMR spectra, 32 transients were accumulated with ^1^H pulse length of 9.10 μs and 5 s relaxation delay, 2.044 s acquisition time, 32,768 date points and 0.30 Hz line broadening. Two-dimensional NOE or NOESY experiments were carried out using 500 ms mixing times, for the detection of a build-up of NOEs. 2D NOE spectra were recorded at 25 °C and 47 °C. ^1^H chemical shift values were referred to DSS as an external reference. The spectra were processed with the use of TopSpin 3.1 (Bruker, Karlsruhe, Germany) software. Apparatus error was ±0.001 ppm.

## 4. Conclusions

TEM studies permitted the characterization of the shape, morphology and size of the examined drug-loaded liposomal nanostructures. The conducted temperature analysis of symmetrical and antisymmetrical methylene stretching bands allowed assessment of the thermotropic phase behavior of the analyzed liposomes, and an estimation of the degree of the membrane ordering. The obtained data are consistent with the results obtained by DSC. Application of FT-IR and NMR allowed exploring mutual interactions between the analyzed drugs and its phospholipid carriers. The resulting degree of encapsulation of cytarabine and methotrexate in liposomes is satisfactory and provides a good basis for further studies of the obtained TDS.

## Figures and Tables

**Table 1:** Pre-transition temperature (Tp) and main transition temperature (T_C_) determined for L_DPPC_ and L_DPPC/AraC/MTX_ liposomes by DSC and FT-IR method.

Liposomes	Phase Transition Temperature (°C)			
	DSC		FT-IR	
	Tp	T_C_	Tp	T_C_
L_DPPC_	34.64 ± 0.14 *	41.30 ± 0.08 *	nd	41.28 ± 0.06 *
L_DPPC/AraC/MTX_	36.39 ± 0.12 *	41.81 ± 0.09 *	36.02 ± 0.11 *	41.95 ± 0.08 *

nd—not detected; * Standard error.

**Table 2:** Assigned bands of the FT-IR spectra of L_DPPC_, L_DPPC/AraC_, L_DPPC/MTX_, L_DPPC/AraC/MTX_ liposomes.

Assignment *	Wavenumber (cm^−1^)			
	L_DPPC_	L_DPPC/AraC_	L_DPPC/MTX_	L_DPPC/AraC/MTX_
ν_as_ (CH_3_)	2955.5	2956.0	2956.0	2956.0
ν_as_ (CH_2_)	2919.0	2918.5	2918.5	2919.0
ν_s_ (CH_2_)	2850.0	2850.5	2850.5	2850.5
ν_as_ (C=O) for ester	1734.5	1732.0	1738.0	1738.0
ν_as_ (PO_2_^−^)	1214.5	1215.0	1222.0	1219.5
ν_s_ (PO_2_^−^)	1085.0	1087.0	1088.5	1089.0
ν_as_ (N^+^-CH_3_)	970.0	970.0	971.5	972.0
γ_r_ (CH_2_)	nd	nd	721.5	721.5

* ν_s_—symmetric stretching vibration; ν_as_—antisymmetric stretching vibration; γ_r_—rocking vibration; nd—not detected.

## Supplementary Materials

Supplementary materials can be accessed at: http://www.mdpi.com/1420-3049/21/12/1689/s1.
