# Supplementary material for: Methotrexate and Cytarabine—Loaded Nanocarriers for Multidrug Cancer Therapy. Spectroscopic Study
Source: Molecules. 2016 Dec 8;21(12):1689. doi: 10.3390/molecules21121689 (PMC6273386; doi:10.3390/molecules21121689)
Supplement: Supplementary file 1 [file molecules-21-01689-s001.pdf]

Supplementary Materials: Methotrexate and Cytarabine—Loaded Nanocarriers for Multidrug Cancer Therapy. Spectroscopic Study

Danuta Pentak, Violetta Kozik, Andrzej Bąk, Paulina Dybał, Aleksander Sochanik and Josef Jampilek

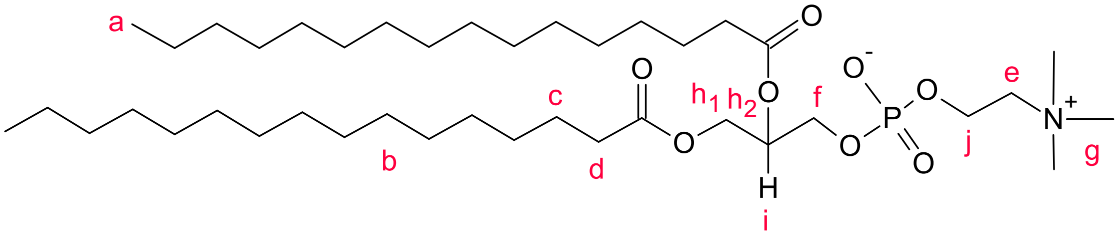

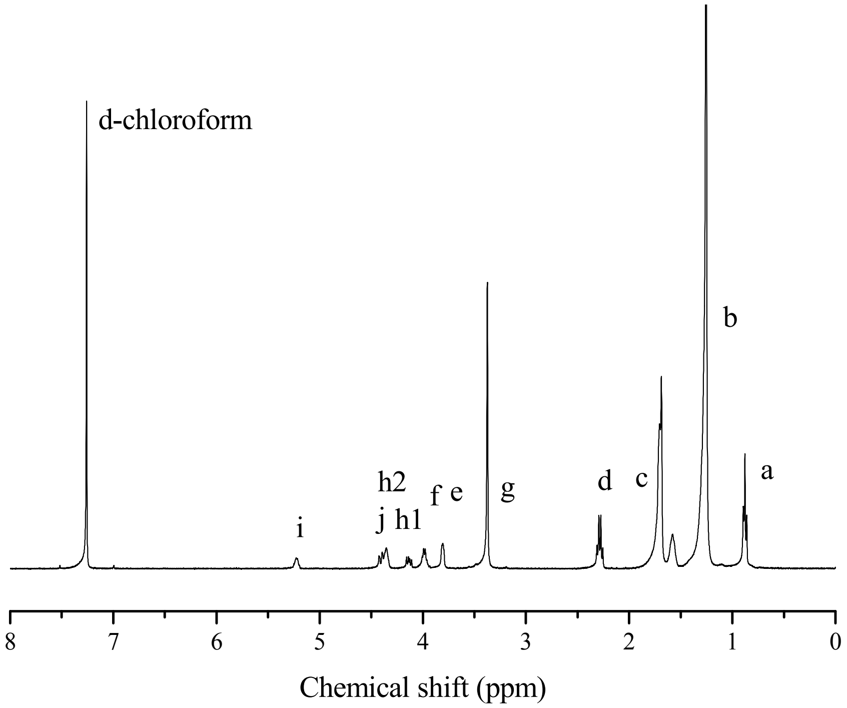

Figure S1. ^{1}H-NMR spectrum of pure DPPC.
